# Measurement of craving among gamers with internet gaming disorder using repeated presentations of game videos: a resting-state electroencephalography study

**DOI:** 10.1186/s12889-023-15750-4

**Published:** 2023-05-04

**Authors:** Sangin Park, Jihyeon Ha, Wonbin Ahn, Laehyun Kim

**Affiliations:** 1 Industry-Academy Cooperation Team, Hanyang University, 222, Wangsimni-ro, Seongdong-gu, Seoul, 04763 South Korea; 2 Center for Bionics, Korea Institute of Science and Technology, 5, Hwarang-ro 14-gil Seongbuk-gu, Seoul, 02792 South Korea; 3 Applied AI Research Lab, LG AI Research, 128, Yeoui-daero, Yeongdeungpo-gu, Seoul, 07796 South Korea; 4 Department of HY-KIST Bio-convergence, Hanyang University, 222, Wangsimni-ro, Seongdong-gu, Seoul, 04763 South Korea

**Keywords:** Internet gaming disorder, Electroencephalography, Repetitive stimulations, Behavior addiction, Craving, Resting-state

## Abstract

**Background:**

Internet gaming disorder (IGD) is receiving increasing attention owing to its effects on daily living and psychological function.

**Methods:**

In this study, electroencephalography was used to compare neural activity triggered by repeated presentation of a stimulus in healthy controls (HCs) and those with IGD. A total of 42 adult men were categorized into two groups (IGD, *n* = 21) based on Y-IAT-K scores. Participants were required to watch repeated presentations of video games while wearing a head-mounted display, and the delta (D), theta (T), alpha (A), beta (B), and gamma (G) activities in the prefrontal (PF), central (C), and parieto-occipital (PO) regions were analyzed.

**Results:**

The IGD group exhibited higher absolute powers of D_C_, D_PO_, T_C_, T_PO_, B_C_, and B_PO_ than HCs. Among the IGD classification models, a neural network achieves the highest average accuracy of 93% (5-fold cross validation) and 84% (test).

**Conclusions:**

These findings may significantly contribute to a more comprehensive understanding of the neurological features associated with IGD and provide potential neurological markers that can be used to distinguish between individuals with IGD and HCs.

## Background

Internet gaming disorder (IGD) is generally defined as problematic and compulsive use of internet gaming, leading to significant impairment in social, educational, and/or occupational activities. IGD has emerged as a social problem in adolescents and young adults because of its high prevalence and various possible comorbidities [1, 2]. Additionally, IGD was included in Section III of the Diagnostic and Statistical Manual of Mental Disorders (DSM-5) and has been classified as a tentative disorder, warranting further research, and is fully recognized as an independent clinical disorder [3]. In 2019, gaming disorder (GD) was defined as a mental illness according to the 11th revision of the World Health Organization’s International Classification of Diseases (ICD-11) [4]. Furthermore, IGD was classified as being most similar to pathological gambling (or “gambling disorder”) by the DSM-5 and was defined as including the following nine criteria [5, 6]: (1) preoccupation with internet games (IG); (2) withdrawal symptoms from IG; (3) tolerance of and increasing engagement in IG; (4) unsuccessful attempts to stop or reduce IG; (5) loss of interest in other hobbies or activities; (6) gameplay (e.g. binge and continuous excessive gaming); (7) deception regarding the amount of time spent engaged in IG; (8) escape or relief from a negative mood; and (9) jeopardized or lost relationship, job, educational or career opportunity. Other studies have defined IGD as engaging in IG for over 14 hours per week for a minimum of one year [7] and reported IG as a major online activity [8].

Internet gaming is a popular and enjoyable activity. However, IGD has received increasing attention because of its negative effects on job and academic performance, normal daily life, and social and psychological functioning [3, 9]. Furthermore, IGD is known to be strongly associated with various comorbid psychological states, such as depressed mood [10] and anxiety [10, 11], psychiatric disorders [12], attention deficit hyperactivity disorder [10, 11], and obsessive-compulsive disorder [10, 13]. Based on 37 cross-sectional studies, IGD is a serious issue worldwide, with a 1.4% prevalence in Norway (among 16–74-year-olds), 1.6% in seven European countries (14–17-year-olds), 4.3% in Hungary (among 15–16-year-olds), 5.5% in Germany (among 13–20-year-olds), 8.5% in the United States (among 8–18-year-olds), 17% in Iran (14–15-year-olds) [14], and 5.9% in South Korea (among 14–15-year-olds) [15]. Evidently, IGD is not a problem confined to individuals but a grave social issue that threatens society. Therefore, research dedicated to relieving this condition is required.

First, an accurate and robust method for measuring symptoms in individuals with IGD should be developed; however, the lack of consistency in screening tools is a major issue in this field [16]. Consequently, several studies have tried to measure IGD characteristics using self-reporting, behavioral responses, physiological responses, and brain function tests, as follows. (1) Several studies have proposed that a GD (or addiction) can be measured by self-reporting using the internet addiction test (IAT) [17], internet-related problem questionnaire [18], 7-item game addiction scale [19], and the video game addiction test [20]. (2) Research into behavioral responses has found that individuals with IGD exhibit a decreased eye blinking rate and saccadic movement [21] and an increased number of regressions in eye movement [22]. (3) Some studies have shown that IGD is associated with physiological responses such as skin conductance responses [23], respiratory rate changes [21], reduction in the standard deviation of normal-to-normal intervals [21] and high frequency of HRV [22, 24, 25]. (4) Other studies have identified the IGD phenomenon in electroencephalogram (EEG) oscillations, brain activity, event-related potentials (ERPs), and activity in functional magnetic resonance imaging (fMRI). Participants with IGD show increasing delta and theta powers in the frontal area [23] and decreasing beta and gamma bands in the frontal and parietal lobes [26, 27]. In addition, the left and right dorsolateral prefrontal cortex (DLPFC), superior parietal lobe, and paralimbic and orbital frontal lobes are negatively associated with response inhibition performance in individuals with IGD [28, 29]. However, positive correlations have been observed in the activity of the prefrontal cortex, anterior cingulate cortex, inter-hemispheric insula connectivity, right inferior temporal cortex, primary somatosensory cortex, inferior parietal lobule, middle occipital gyrus, and bilateral DLPFC [28, 30, 31]. In an ERP study associated with inhibition function, participants with IGD showed that N2 latency at the central [32] and P3 latency at the midline centro-parietal areas [33] were delayed. The N2 amplitude in the frontal area [32] and late positive potential (LPP) in the centro-parietal area [21] increased, and P3 amplitudes at the midline centro-parietal [33–35] and N1 amplitudes in the midline fronto-central regions [35] decreased compared with those in healthy controls (HCs). In fMRI studies, participants with IGD showed higher levels of activation in the lateral and prefrontal cortex, posterior cingulate cortex, right medial orbitofrontal cortex, bilateral supplementary motor area, superior frontal gyrus, inferior frontal gyrus, precentral gyrus, temporal gyrus, left postcentral gyrus, striatum, precuneus, putamen, pallidum, left anterior cingulate (ACC), and left caudate than those in the HCs group, which was related to craving experience, risk-evaluation network, goal-directed behavior, default mode network, cognitive control network, and executive functions [29, 36–40]. These studies used stimuli or tasks to measure the responses of the IGD group, including the resting state [9, 25, 27, 33, 37, 41], playing online or video games [22, 24], watching online gameplay videos or images [36], viewing game videos or images [28, 30, 42, 43], Go/No-Go task [32, 38], cue reactivity task [21, 29], probability discounting task [38], oddball task [33–35], and Stroop task [31, 39].

Several previous studies have investigated the cortical activity associated with IGD to help diagnose, prevent, or treat the condition. However, these studies mainly attempted to confirm activation of the cortex in IGD based on non-repeated stimuli, including playing games and viewing game videos or images. However, they did not examine the physiological responses to repeated stimuli. Some researchers contend that exposure to repeatedly presented stimuli over a short or long period may lower the thresholds of the visual sensory system, partly because of habituation effects [44–46]. However, the psychophysiological responses caused by repetitive stimuli should be investigated when examining the EEG correlates of specific diseases such as unconscious addiction symptoms. Such addiction symptoms may arise from long-term exposure to visual or auditory stimulation via both conscious and unconscious pathways. Synaptic actions are activated by trains of neural impulses, and subtle but reliable changes can be observed at the cortical level. Repeated stimulation more reliably produces strong cortical activity than single stimulation in which information is processed simultaneously along both conscious and unconscious pathways. In addition, dual processing in the cognitive control system reflects habituation and sensitization. Salient stimuli elicit both processes, and electrophysiological outputs reflect the summation of these processes. It has also been reported that cumulative physiological responses to repeatedly presented stimuli do not decrease in participants but rather increase [45, 47]. The tendency toward habituation responses is related to the presence of numerous identical repetitions, whereas sensitization responses present progressively stronger activation in response to repeated stimuli [48]. We considered that physiological responses to repeated stimuli in individuals with IGD might differ significantly from those in HCs because disorders or addictions are affected by differential habituation and sensitization between HCs and IGD groups in response to repeated or prolonged presentation of a stimulus [49, 50]. Thus, we investigated the EEG correlates of IGD using repeatedly presented stimuli, which can induce more robust cortical activity in IGD groups than in previous methods.

There are many previous studies on IGD based on resting state EEG [9, 25, 27, 34, 37, 41]; however, there are few studies regarding its use for IGD measurement or other practical uses in this context. Quantitative measurement of IGD can have positive effects. Accordingly, this study aims to classify participants into HCs and IGD groups by conducting various machine-learning algorithms. In order to better distinguish them, we determined the effect of repeated presentation of a stimulus (gameplay video) on HCs and IGD groups, based on their EEG responses. The hypothesis was as follows: viewing gameplay video could induce different neural activities in individuals from the HCs and the IGD group. As previously mentioned, these activities can be correlated to habituation and sensitization. In this study, we determined the spectral power as features of resting state EEG. We also hypothesized that the whole brain area and all frequency bands were subject to analysis because many previous studies on resting state EEG have reported differences in several frequency bands on various areas. The whole brain was divided into three areas: prefrontal, central, and parieto-occipital. The frequency bands included delta, theta, alpha, beta, and gamma bands.

## Methods

### Participants

Sixty-two male adolescents ranging in age from 14 to 22 years (mean age, 19.31 ± 2.51 years) were recruited for this study. Internet-related disorder has emerged as a social problem in male adolescents, who tend to invest more time playing games than their female peers [51–53]. Therefore, only male adolescents were included in this study. They were required to respond to questionnaire items that measured IGD symptoms using the Korean version of Young’s Internet Addiction Test (IAT) [54], a translated and validated Korean version of the Internet Addiction Test (Y-IAT-K). According to the Y-IAT-K, 42 participants were assigned to two groups, as follows: (1) 21 HCs, ranging in age from 15 to 22 years (mean age, 19.65 ± 2.58 years), with a score of 40 or less and (2) 21 participants with IGD, ranging in age from 14 to 22 years (mean age, 18.80 ± 2.33 years), with a score of 60 or above. Twenty participants who did not meet the inclusion criteria (Y-IAT-K score of 41–59) were excluded from this study. We used the independent sample t-test to confirm that there were no demographic differences in terms of age between the two groups (*t* [39] = − 0.936, *p* = 0.355). We investigated the average gameplay hours per day for 1 week before the experiment in all participants (HCs: 1.02 ± 1.46, IGD: 5.52 ± 2.95). Two-way analyses of variance (ANOVA) was applied in this study and required a sample size of 40 based on G*power calculations (ANOVA: Repeated measures, between factors; effect size *f* = 0.40, *α* = 0.05, 1 − *β* = 0.80, number of group: 2, number of measurements: 2, corr among rep measures: 0.5). This study satisfied the sample size criteria with 84 samples (pre- vs. post-resting state [within-participant factors], 42 samples; IGD group vs. HCs [between-participants factors], 42 samples).

Participation was voluntary, and participants were paid US$ 53.07. All participants were right-handed and had no family or medical history of central nervous system disease. By self-reported questionnaire including asking “Do you have any psychiatric disorder, including other addiction?”, we screened other psychiatric disorders out, such as anxiety, attention deficit hyperactivity disorder (ADHD), obsessive-compulsive disorder, or substance/behavioral addictions. Especially, we tested their rate of anxiety, ADHD, depression, impulsiveness, and aggression by the Korean-Beck Anxiety Inventory, Conners-Wells’ Adolescent Self-Report Scale or Conners Adult ADHD Rating Scales-Self Report, Korean-Beck Depression Inventory-II, Barratt Impulsiveness Scale version 11, Aggression Questionnaire-Korean version, respectively. Finally, HCs had no psychiatric disorder and IGD group also had only IGD. The HCs had previously experienced the games used in this study at least once but did not enjoy the game. The IGD group preferred the games used in this study and enjoyed playing them. They were required to abstain from game-play, alcohol, smoking, and caffeine for at least 24 hours before the experiment and sleep according to their normal schedule. Each participant was informed of the experimental procedure (but not of the research purpose) before providing written informed consent. The experiments were conducted in accordance with the Declaration of Helsinki, and all protocols in this study were approved by the institutional review board.

### Stimuli and experimental procedure

**Fig. 1:**
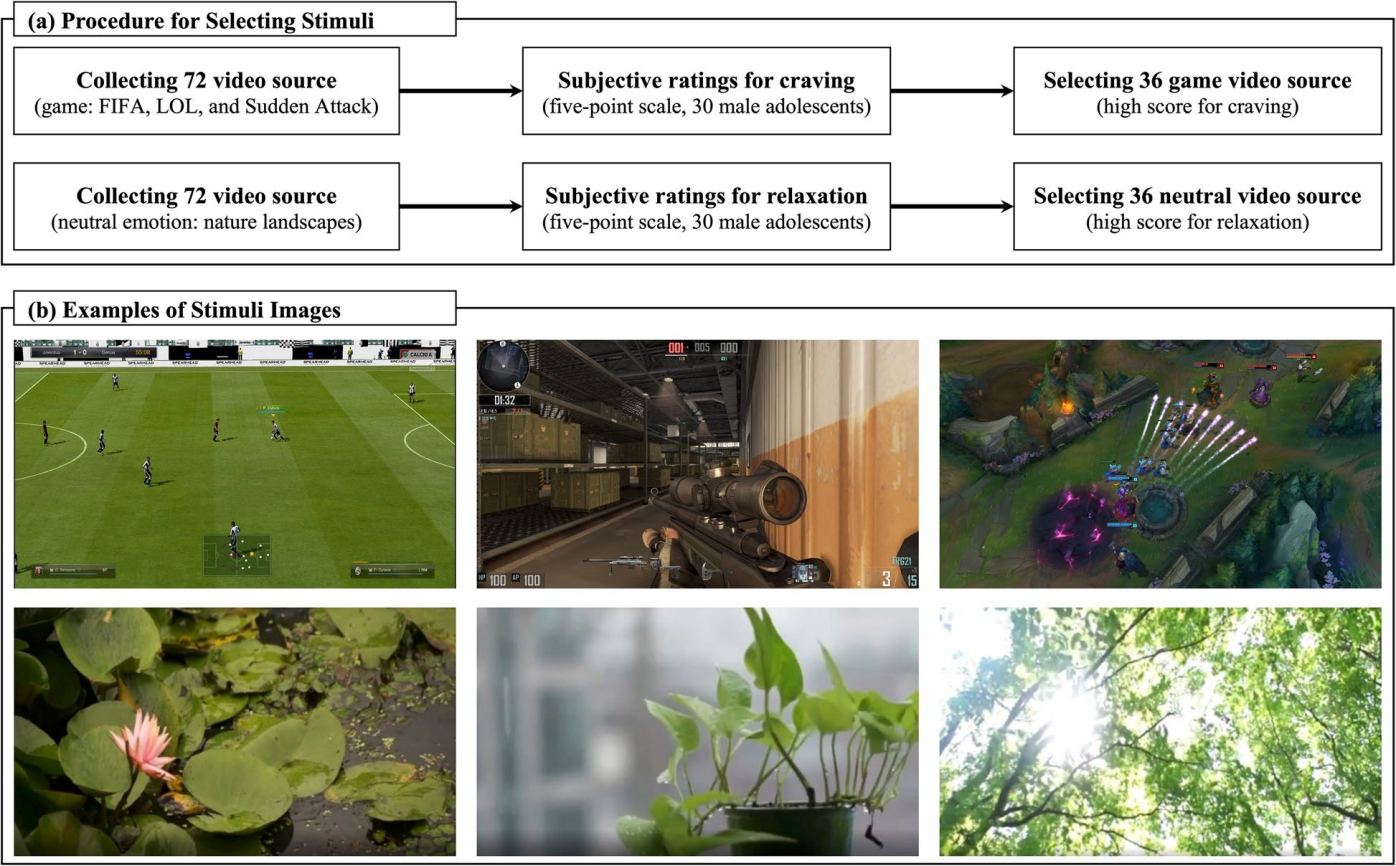
**a** Procedure for stimuli selection. **b** Examples of still images from the game (top; FIFA Online 3, Sudden Attack, and League of Legends) and neutral (bottom) videos

Game videos were used in this study to increase the craving experience for internet gaming. The steps for selecting the stimuli are shown in Fig. 1 and described below. (1) First, as stimuli, we selected three top-ranking games from the best online games in 2017 based on online game ranking in Korea (Naver Inc., Korea), including the League of Legends (Riot Games Inc., USA), Sudden Attack (Nexon Inc., Korea), and FIFA Online 3 (Electronic Arts Inc., USA). We collected 72 video sources of game highlights and fantastic play scenes with numerous hits. (2) We also amassed 72 neutral emotion videos associated with natural landscapes from an internet survey to recover the response to the game stimulus presented above. (3) Game and neutral stimuli were used to survey their suitability, based on subjective ratings (5-point scale), of 30 male adolescents (19.63 ± 2.33 years) for craving and relaxation experience, respectively. Following the subjective ratings, 72 stimuli (36 games and 36 neutral videos) were selected based on their high craving and relaxation scores, as shown in Fig. 1. Subsequently, we recruited participants who enjoyed the game used in the experiment and excluded those who mainly enjoyed other games. Therefore, before the experiment, the participants were controlled such that there was no difference in their familiarity with the videos. The videos selected for this study are available at https://youtu.be/5JL3DVmXaJk.

**Fig. 2:**
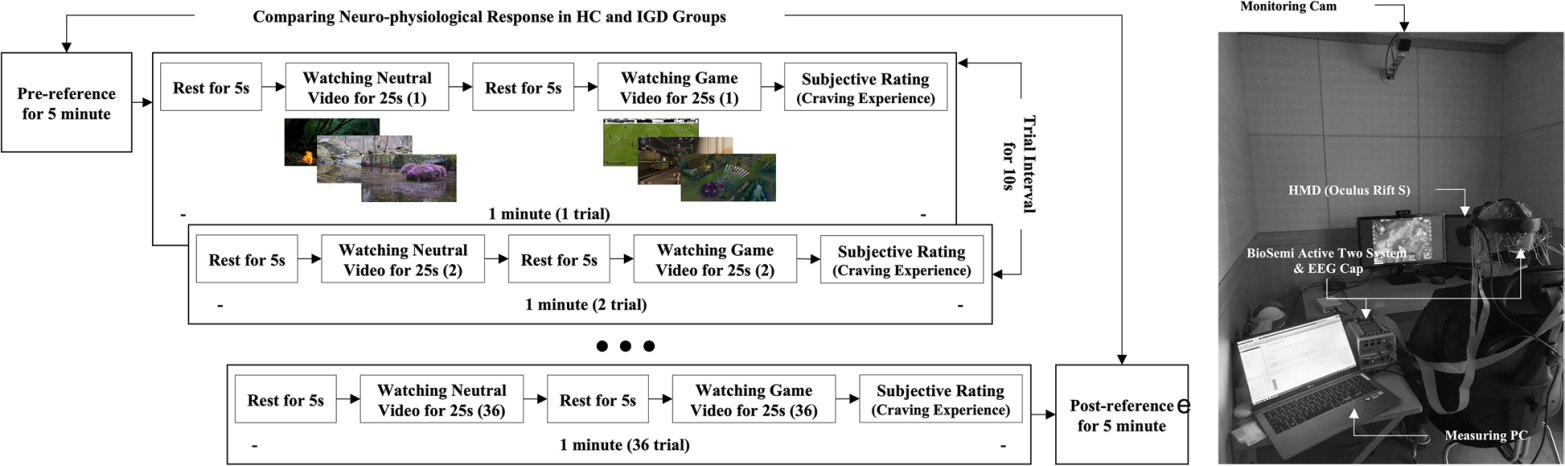
Experimental procedure (left) and environment (right)

Figure 2 illustrates the experimental procedure and environment. The participants watched the video stimuli using head-mounted display virtual reality (VR) devices (Oculus Rift S, Oculus VR Inc., USA) to increase their engagement with the content while sitting on a comfortable chair. Thus, we tested the data in all channels using a simple test before the experiment. No abnormalities in the EEG signals have been reported. The experiment consisted of watching neutral and game videos for 25 s each and resting for 5 s between viewing each stimulus image (one trial). After viewing the game video in each trial, the participants were required to respond to their craving experience (5-point scale) to play a game. In this experiment, 36 gameplay videos (three types of games × 12 examples) and 36 neutral videos were used. We applied counterbalancing to the game types. As there were 12 different videos for each game type, the videos could be arranged randomly such that runs of the same type did not occur. In addition, 36 neutral videos were randomly selected. Each trial consisted of watching one neutral video and one video game. The various stimulus sets consisted of 36 trials organized in this manner. Finally, we randomly selected one of the stimulus sets to use with a given participant. The trial intervals lasted 10 s, and the pre- and post-resting states were in a relaxed environment for 5 min each before and after the experiment. EEG signals were measured during the experiment. As this study aimed to investigate the effect of the habituation process by repeated presentation of a stimulus on the HCs and IGD groups, EEG activity was analyzed in the pre- and post-resting states for 5 min each. Spectral EEG activity (i.e., power ratio) in the post-resting state was compared to subtle changes recorded in the pre-resting state. EEG data recorded during observation of gaming and neutral videos were not included in the data analysis.

### Data acquisition and signals processing

The EEG data were recorded at a sampling rate of 2048 Hz from 64 channels mounted on an EEG electrode cap (Active-two, BioSemi S.V., Amsterdam, Netherlands) arranged in the international 10–20 montage, and the ground and reference electrodes were replaced by the common mode sense (CMS)/driven right leg (DRL), which is specific to BioSemi systems (cf. http://www.biosemi.com/faq/cms&drl.htm for further information). To analyze the EEG signals, they were downsampled to 512 Hz and re-referenced using a common average referencing (CAR) procedure. CAR was calculated by subtracting each channel from the average potential over all channels at each time step. This is also known as removing the global background activity and maintaining the activity from local sources beneath the electrodes [55], whereas other resting-state EEG studies have applied the CAR procedure [56]. The EEG signals were then processed using a band-pass filter (Butterworth type of order six) of 0.5–55 Hz. However, because EEG channels can be contaminated by noise such as ocular and muscular artifacts, these artifacts were removed from the EEG signals using artifact subspace reconstruction [57]. Among the total 5 min pre- and post-resting state data, we used only the first part, 2 min data for calculating power spectral density (PSD). The PSD was analyzed using Welch’s method and the parameters were as follow: window size, 5 seconds; window overlap size, 1 seconds; frequency resolution, 0.2 Hz. The EEG spectrum was divided according to the frequency band into the following ranges: delta (D) 1–4 Hz, theta (T) 4–8 Hz, alpha (A) 8–13 Hz, beta (B) 13–30 Hz, and gamma (G) 30–50 Hz [58, 59]. Brain regions in this study were defined as the prefrontal (FP_1_, FP_Z_, FP_2_, and AF_Z_), central (C_3_, C_1_, C_Z_, C_2_, and C_4_), and parieto-occipital areas (PO_3_, PO_Z_, PO_4_, and O_Z_), and the absolute powers from the averaged signals in each brain region were extracted. The EEG signals were measured before, during, and after stimulus presentation. However, the EEG data recorded during the observation of game and neutral videos were not included in the data analysis because this study aimed to investigate subtle changes in EEG activity between the pre- and post-resting states in the HC_S_ and IGD groups. All signal processing and data analyses were performed using EEGLAB, a toolbox in MATLAB Mathworks Inc., Natick, MA, USA).

### Statistical analysis

This study was designed to compare changes in cortical activity between the pre- and post-resting states (within-participants factor) between the IGD and HC_S_ groups (between-participants factor). Next, ANOVA was performed to investigate group-by-time interaction effects and each main effect on the EEG spectral power. Comparisons of subjective ratings (i.e., craving scores) between the HC_S_ and IGD groups were performed using the Mann–Whitney U test with a smaller sample size. To confirm practical significance, the effect size was calculated based on a two-way ANOVA (partial eta-squared, ƞp^2^) and Mann–Whitney U test (r). The standard partial eta-squared (ƞp^2^) values of 0.01, 0.06, and 0.14 for effect size are generally regarded as small, medium, and large, respectively [60]. In the case of non-parametric tests (absolute value of r), standard values of 0.10, 0.30, and 0.50 for effect size are generally considered small, medium, and large, respectively [61]. The expected effect size as a partial eta-squared value (ƞp^2^) was 0.087 (α = 0.05, 1 – β = 0.80, and total sample size = 84), as calculated by G*power software. To correct inflated type I errors caused by multiple comparisons, statistical significance was adjusted using the Benjamini–Hochberg (BH) false discovery rate (FDR) correction [62]. BH correction controls *p*-values more effectively than the traditional Bonferroni correction [62]. Additionally, the BH correction has been applied in many neurophysiological studies to handle the increasing rate of type I errors caused by multiple null hypothesis testing in a statistically valid manner [63]. In this study, the alpha level of 0.05 was used as the FDR criterion. As shown in the Results section, the p-value for the prefrontal theta was the highest p-value smaller than the critical value (0.0228 < 0.0233). All values above this value (i.e. those with lower p-values) were considered significant. Therefore, the adjusted alpha level was 0.0228 after BH. Thus, we conducted a correlation analysis between significant EEG features, the Y-IAT-K, and craving scores using Spearman’s rank correlation. All statistical analyses were conducted using IBM SPSS Statistics 21.0, for Windows (SPSS Inc., Chicago, IL, USA).

### Classification

To determine the best classification algorithm for our features, we used three basic machine learning algorithms: Discriminant Analysis (DA), Support Vector Machine (SVM), and Neural Network (NN) [64–67]. To train the above classifiers, the Classification Learner App from the MATLAB toolbox (2022b, Mathworks Inc.) was used. We obtained the parameters for each classifier using the hyperparameter optimizer in the classification learner application. The optimization options included: optimizer, Bayesian optimization; acquisition function, expected improvement per second plus; iterations, 100; and training time limit, no. The classifiers were validated 5-fold cross-validation with cross-validated portion including shuffled samples and tested with tested portion 0.3. We trained the above classifiers with optimized parameters. In this study, the number of features for classifiers were 15. And the number for total samples were 168 by augmenting samples. Total samples were randomly allocated to training, validation and test by using ‘cvpartition’ function with hold out option (0.3) and kfold option (5-fold). Of these samples, 120 were allocated to training and validation, while the remaining 48 were designated as test samples. Sample augmentation was conducted by dividing 5-minute data into 2-minute segmentation was conducted by dividing 5-minute data into 2-minute segments with 50% overlap, and ensuring that the training, validation, and test samples did not include data from the same subjects. Validation accuracy was calculated as the average accuracy for 5-fold cross-validated data, while test accuracy was determined as the average accuracy for test data on each fold. We also reported the area under the curve (AUC) of the ROC curve to represent its performance. Here, AUC is the area under the ROC curve (X-axis: 1 − specificity, Y-axis: sensitivity). The AUC value lies between 0 and 1, where 0 denotes a bad classifier and 1 denotes an excellent classifier [68].

## Results

### Craving scores

**Fig. 3:**
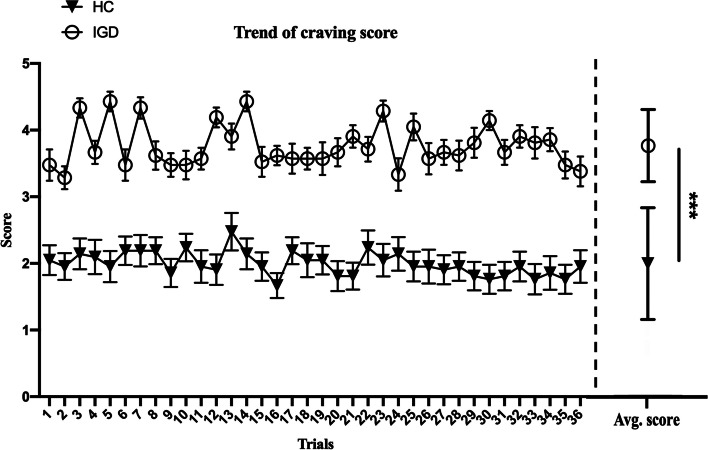
Results of subjective ratings for craving experience (mean and scores from 36 trials) in the healthy controls (HCs) and internet gaming disorder (IGD) groups (*** *p* < 0.001)

The Mann–Whitney U test demonstrated that the subjective rating for craving experience (mean of 36 trials) was significantly higher in the IGD group than in the HC_S_ (3.76 ± 0.84 vs. 1.99 ± 0.54; U = 24.00, *p* < 0.001, *r* = 0.78, with a large effect size). In all trials, craving scores tended to be maintained without any significant differences, whereas both groups experienced repetitive game stimuli. The craving scores of the IGD group were higher than those of the HC_S_ group (Fig. 3).

### EEG activity

**Fig. 4:**
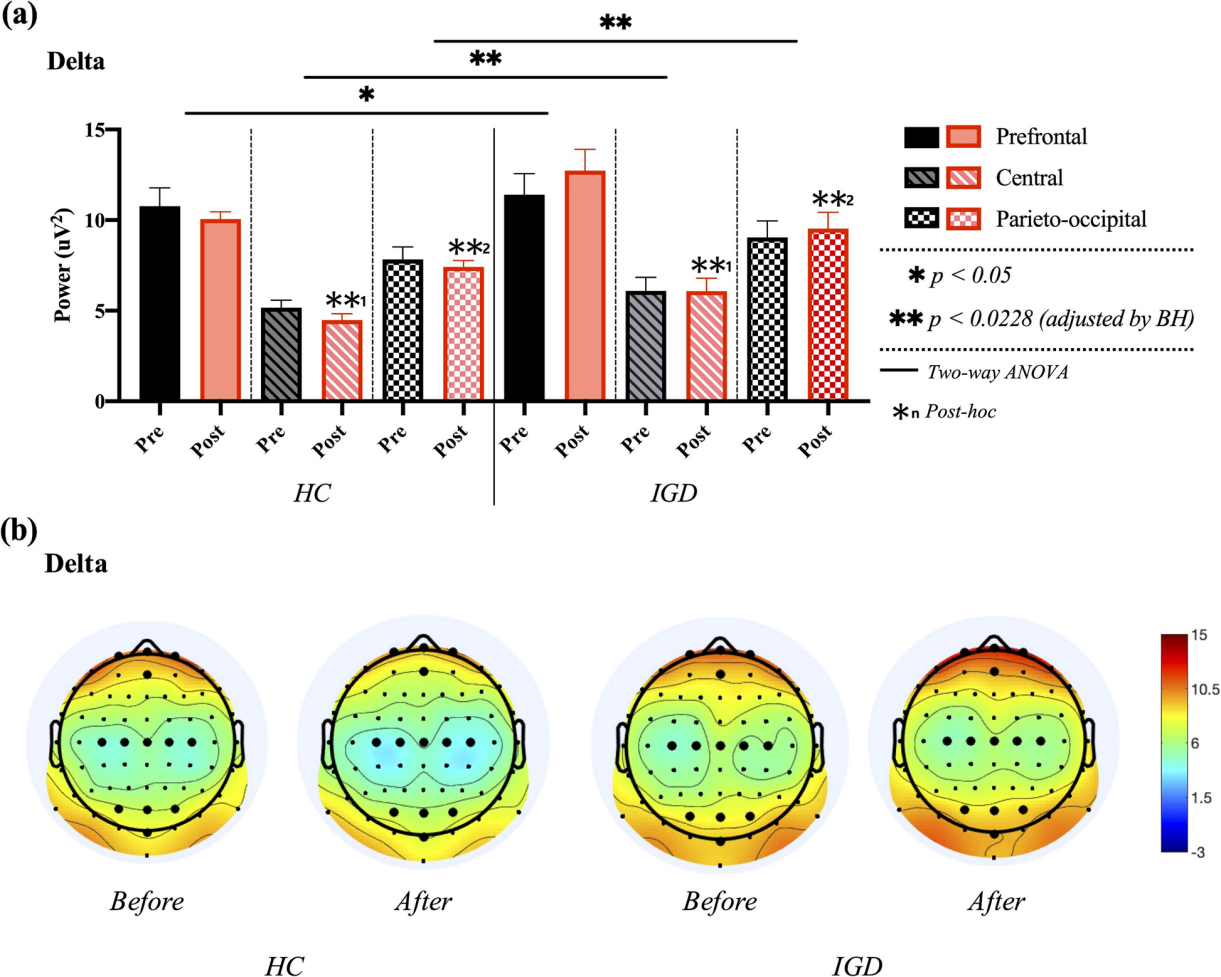
Comparisons of averaged delta power in prefrontal, central, and parieto-occipital regions between healthy control (HC) and internet gaming disorder (IGD) groups in both pre- and post-resting states. **a** Results of two-way ANOVA. **b** Electroencephalogram (EEG) topography in the delta band (1–4 Hz) in pre- and post-resting states

In this study, the EEG features in the brain area were defined by the following abbreviations: (1) delta, theta, alpha, beta, and gamma power in the prefrontal region: D_PF_, T_PF_, A_PF_, B_PF_, and G_PF_, respectively. (2) Delta, theta, alpha, beta, and gamma powers in the central region: D_C_, T_C_, A_C_, B_C_, and G_C_, respectively. (3) Delta, theta, alpha, beta, and gamma powers in the parieto-occipital region: D_PO_, T_PO_, A_PO_, B_PO_, and G_PO_. A two-way ANOVA was used for the entire EEG data set based on the results of the Shapiro–Wilk normality test (*p* > 0.05). Two-way ANOVA for delta power in the prefrontal, central, and parieto-occipital regions revealed a significant main effect of the group in D_C_ (F_1, 38_ = 7.711, *p* = 0.007, ƞp^2^ = 0.089) and D_PO_ regions (F_1, 38_ = 7.031, *p* = 0.010, ƞp^2^ = 0.082), whereas D_PF_ showed no significant difference (F_1, 38_ = 4.079, *p* = 0.049). The main effects of time on D_PF_ (F_1, 38_ = 0.112, *p* = 0.739), D_C_ (F_1, 38_ = 0.472, *p* = 0.494), and D_PO_ (F_1, 38_ = 0.000, *p* = 0.988) were not significant. No significant group × time interaction was identified. In the post-hoc analysis for main effect of group, an independent samples t-test revealed that delta power in the IGD group was significantly larger than that in the HCs group in central (t [39] = − 2.777, p = 0.007) and parieto-occipital regions (t [39] = − 2.695, *p* = 0.009), as shown in Fig. 4.

**Fig. 5:**
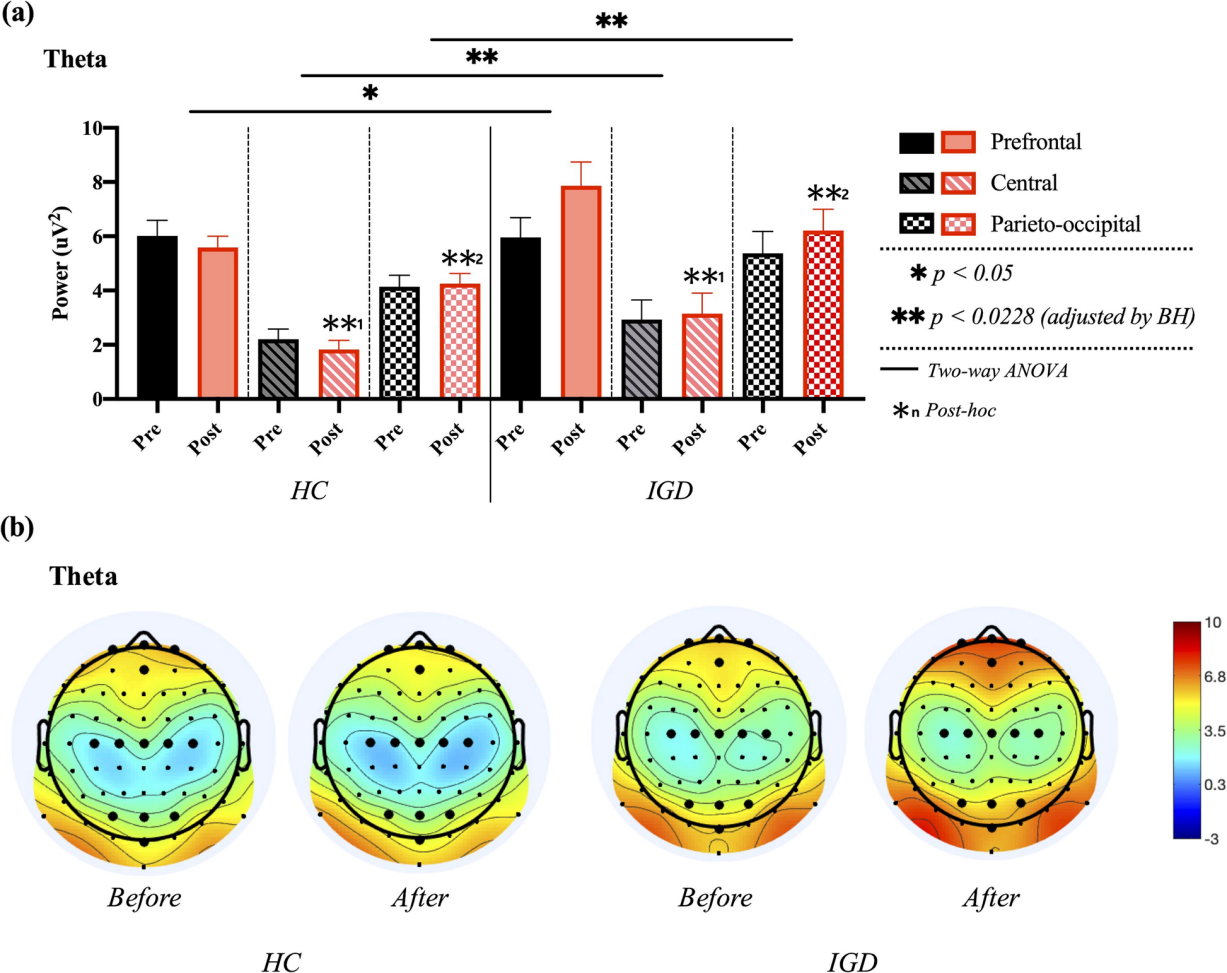
Comparisons of averaged theta power in prefrontal, central, and parieto-occipital regions between healthy control (HCs) and internet gaming disorder (IGD) groups in both pre- and post-resting states. **a** Results of two-way ANOVA. **b** Electroencephalogram (EEG) topography in the theta band (4–8 Hz) in pre- and post-resting states

Two-way ANOVA for theta power in the prefrontal, central, and parieto-occipital regions revealed a significant main effect of the group in T_C_ (F_1, 38_ = 5.506, *p* = 0.021, ƞp^2^ = 0.065) and T_PO_ regions (F_1, 38_ = 10.153, *p* = 0.002, ƞp^2^ = 0.114), whereas T_PF_ showed no significant difference (F_1, 38_ = 5.085, *p* = 0.027, ƞp^2^ = 0.060). The main effects of time on T_PF_ (F_1, 38_ = 1.246, *p* = 0.268), T_C_ (F_1, 38_ = 0.021, *p* = 0.885), and T_PO_ (F_1, 38_ = 0.565, *p* = 0.455) were not significant. No significant group × time interaction was observed. In the post-hoc analysis for main effect of group, an independent samples *t*-test revealed that theta power in the IGD group was significantly larger than that in the HCs group in central (*t* [39] = − 2.415, *p* = 0.019) and parieto-occipital regions (*t* [39] = − 3.184, *p* = 0.002), as shown in Fig. 5.

**Fig. 6:**
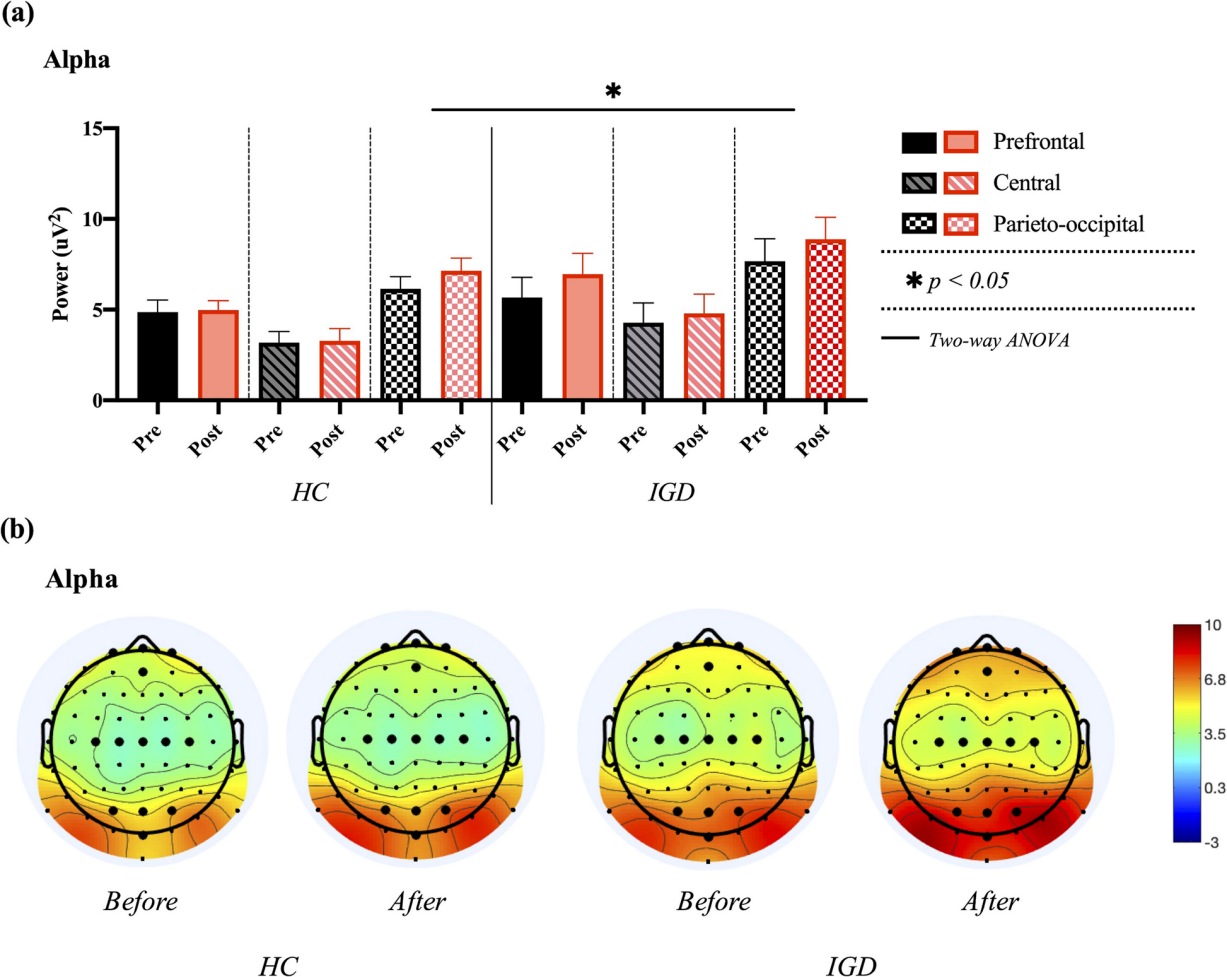
Comparisons of averaged alpha power in prefrontal, central, and parieto-occipital regions between healthy control (HC_)_ and internet gaming disorder (IGD) groups in both pre- and post-resting states. **a** Results of two-way ANOVA. **b** Electroencephalogram (EEG) topography in the alpha band (8–13 Hz) in pre- and post-resting states

Two-way ANOVA for alpha power in the prefrontal, central, and parieto-occipital regions revealed no significant results for the main effects of the group in A_FP_ (F_1, 38_ = 3.698, *p* = 0.058), A_C_ (F_1, 38_ = 3.151, *p* = 0.080), A_PO_ (F_1, 38_ = 4.226, *p* = 0.043), or time in A_FP_ (F_1, 38_ = 0.592, *p* = 0.444), A_C_ (F_1, 38_ = 0.152, *p* = 0.698), and A_PO_ (F_1, 38_ = 1.345, *p* = 0.250). In addition, no significant group × time interaction was found, as shown in Fig. 6.

**Fig. 7:**
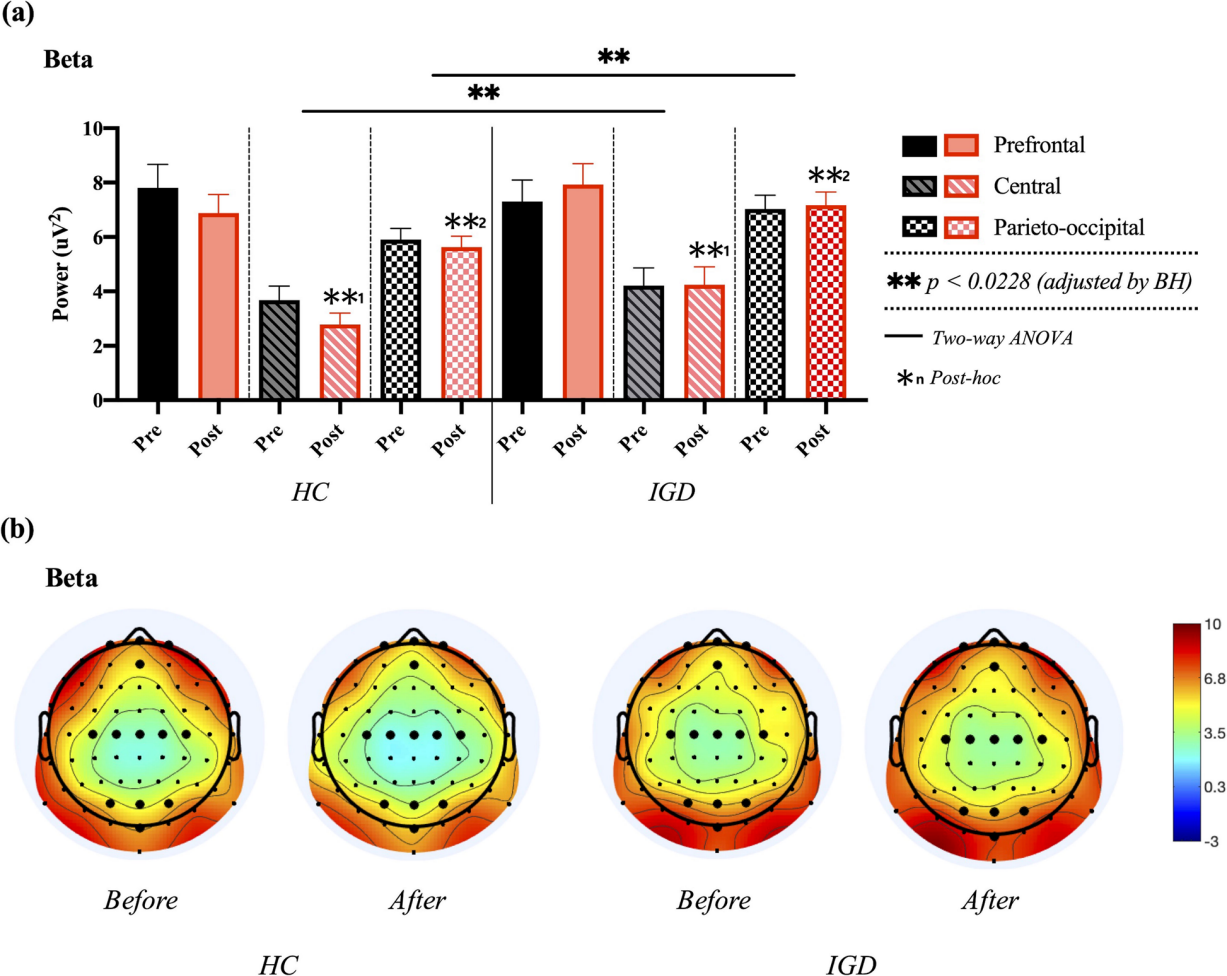
Comparisons of averaged beta power in prefrontal, central, and parieto-occipital regions between healthy control (HC) and internet gaming disorder (IGD) groups in both pre- and post-resting states. a Results of two-way ANOVA. b Electroencephalogram (EEG) topography in the beta band (13–30 Hz) for in pre- and post-resting states

Two-way ANOVA for beta power in the prefrontal, central, and parieto-occipital regions revealed a significant main effect of the group in B_C_ (F_1, 38_ = 5.768, p = 0.019, ƞp^2^ = 0.068) and B_PO_ regions (F_1, 38_ = 10.272, *p* = 0.002, ƞp^2^ = 0.115), but B_PF_ showed no significant results (F_1, 38_ = 0.034, *p* = 0.854). The main effects of time on B_PF_ (F_1, 38_ = 0.017, *p* = 0.897), B_C_ (F_1, 38_ = 0.483, *p* = 0.489), and B_PO_ (F_1, 38_ = 0.051, *p* = 0.822) were not significant. No significant group × time interaction was found. In the post-hoc analysis for main effect of group, an independent samples t-test revealed that beta power in the IGD group was significantly larger than that in the HCs group in central (*t* [39] = − 2.480, *p* = 0.016) and parieto-occipital regions (*t* [39] = − 3.249, *p* = 0.002), as shown in Fig. 7.

**Fig. 8:**
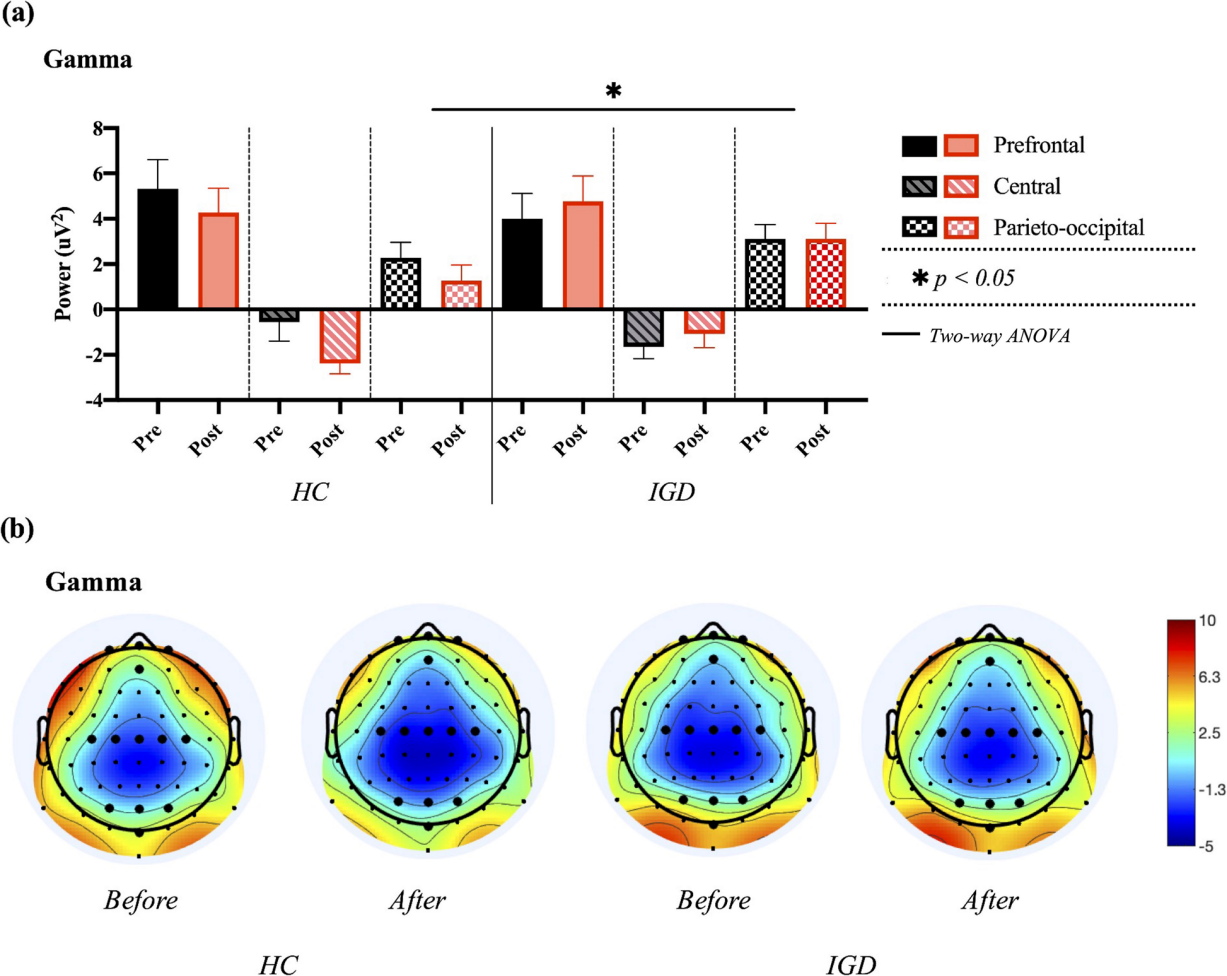
Comparisons of averaged gamma power in prefrontal, central, and parieto-occipital regions between healthy control (HC) and internet gaming disorder (IGD) groups in both pre- and post-resting states. **a** Results of two-way ANOVA. **b** Electroencephalogram (EEG) topography in the gamma band (30–50 Hz) in pre- and post-resting states

Two-way ANOVA for gamma power in the prefrontal, central, and parieto-occipital regions revealed no significant results for the main effect of the group in G_FP_ (F_1, 38_ = 0.378, *p* = 0.540), G_C_ (F_1, 38_ = 0.667, *p* = 0.417), and G_PO_ (F_1, 38_ = 4.151, *p* = 0.045) or time in G_FP_ (F_1, 38_ = 0.010, *p* = 0.922), G_C_ (F_1, 38_ = 0.639, *p* = 0.427), and G_PO_ (F_1, 38_ = 0.578, *p* = 0.450). No significant group × time interaction was found, as shown in Fig. 8. The detailed results are shown in Table 1.

**Table 1:** Summary table of two-way ANOVA. The findings confirmed statistical significance is bolded (*p* < 0.0228)

		***Source***	***Type III Sum of Squares***	***df***	***Mean Square***	***F***	***p***	***Partial Eta Squared***
***Delta***	***PF***	Group	81.632	1	81.632	4.079	0.047	0.049
		Time	2.243	1	2.243	0.112	0.739	0.001
		Group*Time	20.945	1	20.945	1.047	0.309	0.013
	***C***	**Group**	**46.208**	**1**	**46.208**	**7.711**	**0.007**	**0.089**
		Time	2.827	1	2.827	0.472	0.494	0.006
		Group*Time	2.711	1	2.711	0.452	0.503	0.006
	***PO***	**Group**	**70.055**	**1**	**70.055**	**7.031**	**0.010**	**0.082**
		Time	0.002	1	0.002	0.000	0.988	0.000
		Group*Time	4.752	1	4.752	0.477	0.492	0.006
***Theta***	***PF***	Group	45.574	1	45.574	5.085	0.027	0.060
		Time	11.169	1	11.169	1.246	0.268	0.016
		Group*Time	28.545	1	28.545	3.185	0.078	0.039
	***C***	**Group**	**34.008**	**1**	**34.008**	**5.506**	**0.021**	**0.065**
		Time	0.130	1	0.130	0.021	0.885	0.000
		Group*Time	1.845	1	1.845	0.299	0.586	0.004
	***PO***	**Group**	**69.754**	**1**	**69.754**	**10.153**	**0.002**	**0.114**
		Time	3.879	1	3.879	0.565	0.455	0.007
		Group*Time	3.499	1	3.499	0.509	0.478	0.006
***Alpha***	***PF***	Group	63.293	1	63.293	3.698	0.058	0.045
		Time	10.127	1	10.127	0.592	0.444	0.007
		Group*Time	7.551	1	7.551	0.441	0.508	0.006
	***C***	Group	49.393	1	49.393	3.151	0.080	0.038
		Time	2.375	1	2.375	0.152	0.698	0.002
		Group*Time	0.596	1	0.596	0.038	0.846	0.000
	***PO***	Group	79.619	1	79.619	4.226	0.043	0.051
		Time	25.340	1	25.340	1.345	0.250	0.017
		Group*Time	0.279	1	0.279	0.015	0.903	0.000
***Beta***	***PF***	Group	0.442	1	0.442	0.034	0.854	0.000
		Time	0.217	1	0.217	0.017	0.897	0.000
		Group*Time	11.301	1	11.301	0.871	0.354	0.011
	***C***	**Group**	**34.335**	**1**	**34.335**	**5.768**	**0.019**	**0.068**
		Time	2.876	1	2.876	0.483	0.489	0.006
		Group*Time	3.480	1	3.480	0.585	0.447	0.007
	***PO***	**Group**	**39.389**	**1**	**39.389**	**10.272**	**0.002**	**0.115**
		Time	0.195	1	0.195	0.051	0.822	0.001
		Group*Time	1.184	1	1.184	0.309	0.580	0.004
***Gamma***	***PF***	Group	10.650	1	10.650	0.378	0.540	0.005
		Time	0.270	1	0.270	0.010	0.922	0.000
		Group*Time	16.167	1	16.167	0.574	0.451	0.007
	***C***	Group	4.500	1	4.500	0.667	0.417	0.008
		Time	4.310	1	4.310	0.639	0.427	0.008
		Group*Time	22.043	1	22.043	3.267	0.074	0.040
	***PO***	Group	38.533	1	38.533	4.151	0.045	0.050
		Time	5.362	1	5.362	0.578	0.450	0.007
		Group*Time	5.559	1	5.559	0.599	0.441	0.008

### Classification

#### Feature design

In this study, we compared EEG power between the HC_S_ and IGD groups. We calculated and used “Power difference” as a feature for classification to clarify the differences between EEG power in the post-resting and pre-resting states as post-resting EEG after undergoing the experimental protocol affected HC and IGD differently and each participant had different pre-resting state EEG.

$$\boldsymbol{Power}\ \boldsymbol{difference}=\boldsymbol{Post}\ \boldsymbol{EEG}\ \boldsymbol{power}\ \left(\boldsymbol{dB}\right)-\boldsymbol{Pre}\ \boldsymbol{EEG}\ \boldsymbol{power}\ \left(\boldsymbol{dB}\right)$$

The following features were used for classification: power ratio for all frequency bands over the entire brain area (15 features).

#### Performance

**Fig. 9:**
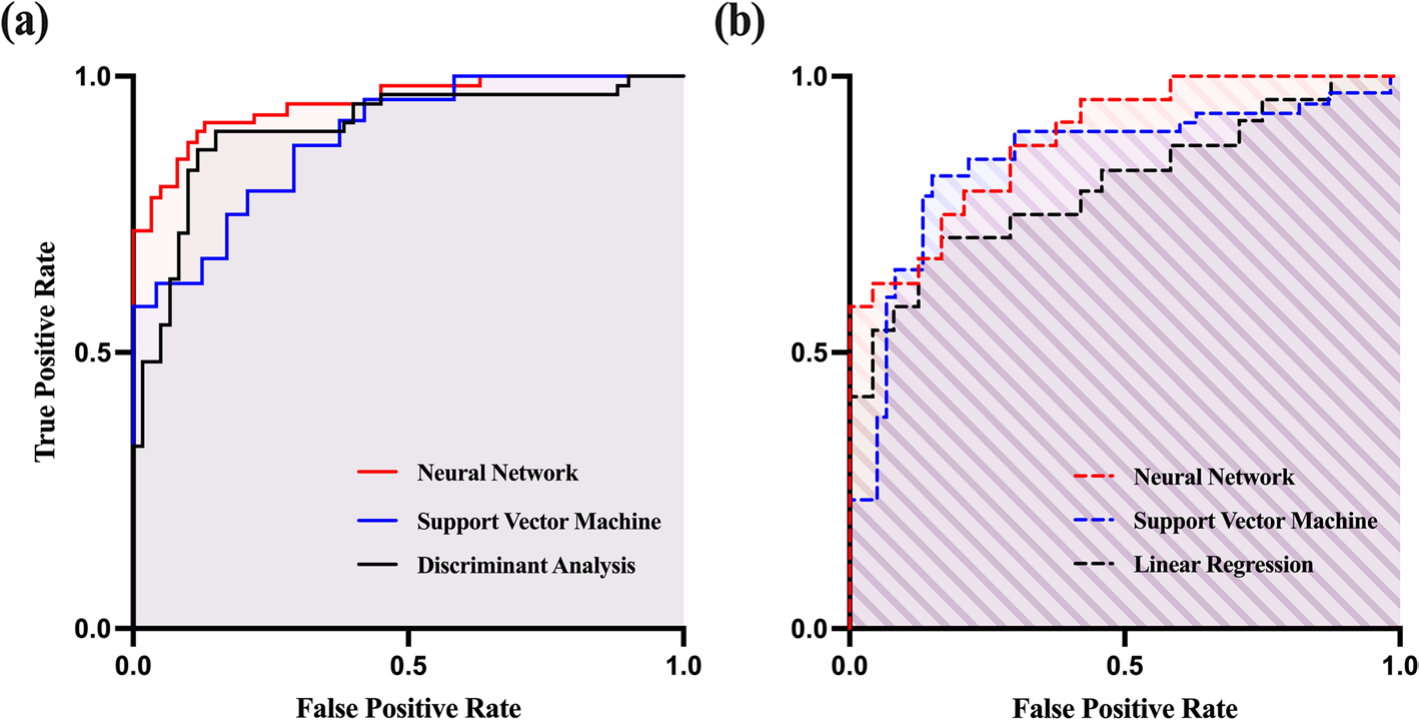
Receiver operating characteristics curves for three classifiers on (**a**) 5-fold cross-validation and (**b**) Test

As shown in Table 2, we classified participants to distinguish between the HC_S_ and IGD groups. According to the three classifiers (DA, SVM, and NN), we achieved the following respective values for accuracy (0.87, 0.86, and 0.93), sensitivity (0.85, 0.89, and 0.96), specificity (0.89, 0.85, and 0.91), and AUC (0.90, 0.89, and 0.94) on 5-fold cross validation. On test data, the performance was respectively accuracy (0.73, 0.75, and 0.84), sensitivity (0.76, 0.77, and 0.82), specificity (0.73, 0.73, and 0.87), and AUC (0.80, 0.85, and 0.89). The ROC curves for three classification methods are shown in Fig. 9.

**Table 2:** Performance of different types of classifiers discriminated by healthy control (HC) and internet gaming disorder (IGD) groups

	Validation				Test			
	***Accuracy***	***Sensitivity***	***Specificity***	***AUC***	***Accuracy***	***Sensitivity***	***Specificity***	***AUC***
***Discriminant Analysis***	0.87 ± 0.04	0.85 ± 0.05	0.89 ± 0.04	0.90 ± 0.03	0.73 ± 0.04	0.76 ± 0.06	0.73 ± 0.09	0.80 ± 0.06
***Support Vector Machine***	0.86 ± 0.05	0.89 ± 0.07	0.85 ± 0.10	0.89 ± 0.04	0.75 ± 0.05	0.77 ± 0.06	0.73 ± 0.05	0.85 ± 0.06
***Neural Network***	0.93 ± 0.06	0.96 ± 0.05	0.91 ± 0.06	0.94 ± 0.03	0.84 ± 0.09	0.82 ± 0.08	0.87 ± 0.12	0.89 ± 0.04

**Fig. 10:**
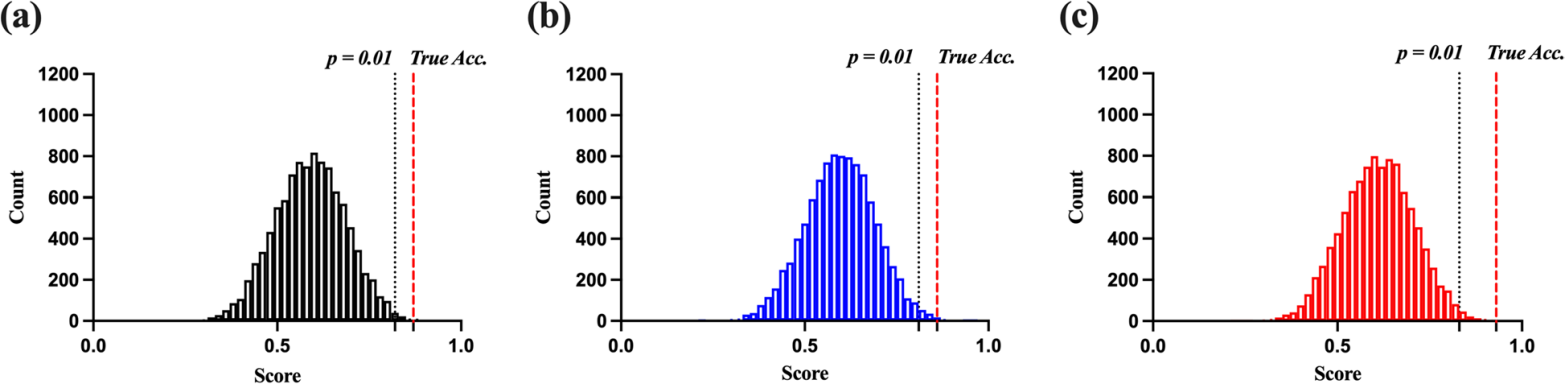
The distribution for the three classifiers for the permutation test (*p* < 0.01). **a** Discriminant Analysis. **b** Support Vector Machine. **c** Neural Network

Permutation tests were conducted for all the classifiers under each condition (10,000 cycles). The results for all feature conditions were as follows: (1) DA: validation accuracy, 0.87; *p* < 0.01; (2) SVM: validation accuracy, 0.86; *p* < 0.01; and (3) NN: validation accuracy, 0.93; *p* < 0.01. Figure 10 shows the distribution of the permutation test

## Discussion and conclusion

The primary aim of this study was to determine the electrophysiological features of individuals with IGD by comparing the EEG activity of the HC_S_ and IGD groups during the repeated presentation of a stimulus (game video). Participants were assigned to the HC_S_ and IGD groups based on their Y-IAT-K scores, and their craving scores for gaming were measured while performing the task. The subjective ratings for craving scores in the HCs and IGD groups indicated that participants in the IGD group experienced cravings for gaming, whereas those in the HCs group did not. We confirmed the results of the subjective rating of the craving experienced by classifying the two groups according to significant differences. EEG features showed significant differences between the IGD and HCs groups. Because these features were significant on two-way ANOVA on group effect, they could distinguish between the HCs and IGD groups, as well. Among the algorithms used to classify the participants into IGD and HCs groups, the NN algorithm demonstrated the highest average recognition accuracy of 93% (5-fold cross-validation) and 84% (test). Hence, NN was found to be the most suitable classifier for the IGD group.

The IGD group showed higher absolute powers of D_C_, D_PO_, T_C_, T_PO_, B_C_, and B_PO_ than the HCs group after repeated presentations of the game stimuli. A two-way ANOVA revealed that there was no statistically significant interaction between the effects of group and time. The main effects of the group showed a significant difference, but time was not significant. These results can be interpreted to mean that the group did have a statistically significant effect on absolute powers of D_C_, D_PO_, T_C_, T_PO_, B_C_, and B_PO_. Previous studies have reported that patients with addictive disorders exhibit flow experiences with feelings of enjoyment arising from deep immersion (high-level attention) [69], dysfunction of behavioral inhibition in the prefrontal areas [21, 33–35]; and activation of the reward circuit (impaired executive control) [23, 41, 70, 71]. The present findings indicate a significant difference in delta, theta, and beta powers between the IGD and HCs groups, which is associated with a high-level attention state, based on previous study results. Delta, theta, and beta oscillations are strongly related to many cognitive processes including attention, memory operations, decision-making and action control, memory recognition, and mental workload in the frontal, central, temporal, and parietal regions [23, 72–74]. In addition, changes in delta and beta activities are associated with behavioral inhibition [75, 76] and reward networks [77, 78], respectively. Thus, the changes in delta, theta, and beta powers found in this study can be interpreted as being strongly related to flow experience, dysfunction of behavioral inhibition, and activation of the reward circuit. Our results provide evidence that supports the potential use of changes in delta, theta, and beta powers as electrophysiological features of the traits investigated in this study, which is consistent with findings from previous studies on patients with addictive disorders as follows: (1) delta, theta [79], and beta powers [79, 80] are increased in gambling disorders; (2) delta [81, 82], theta [81], and beta powers [81, 83] are increased in alcohol use disorders; (3) delta, theta [84], and beta powers [85] are increased in food disorders; (4) beta [86] power is increased in smokers; and (5) theta [87] power is increased in cocaine users.

As mentioned above, because delta and theta powers are strongly related to cognitive processes and behavioral inhibition, HCs can be interpreted as having a low attention level and normal function for behavioral inhibition caused by the repeated presentation of game stimuli. In addition, theta oscillations are likely generated in the ACC and subcortical limbic structures such as the hippocampus [88, 89], and decreasing theta oscillations are associated with low cognitive control [90, 91]. Moreover, decreasing delta oscillations have been reported to be related to mental workload [92, 93]. Thus, the low interest (attention) in the stimuli and high mental workload of the participants in the HCs group indicated that they found repeated presentations of game videos boring and mental stress. The difference between the two groups was related to the degree of interest in the game; therefore, the EEG response to repeated presentation stimuli may be used as an electrophysiological feature to distinguish between the IGD and HCs groups.

Several previous studies related to pure resting-state EEG have reported that subjects suffering from IGD reveal increasing delta and theta powers in the frontal area [23] and decreasing beta and gamma bands in the frontal and parietal lobes [26, 27]. This is inconsistent with the findings of our study. The results of pure resting-state EEG may be different from our findings because disorders or addictions are affected by differential habituation and sensitization between IGD and HCs groups from repeated or prolonged presentation of a stimulus [49, 50]. This study used repeated or prolonged stimulus to induce a distinct difference in the resting-state EEG between the IGD and HCs groups. Repeated stimulation can lead to different habituation or sensitization processes depending on the person’s interest in stimulation, further enhancing cortical activity. Repeated stimulus paradigms generate stronger cortical activity than does a single stimulus. Moreover, repeated stimuli cause cumulative physiological responses to increase [49, 50]. For example, since the HCs group had little or no interest in the game stimuli, the habituation process (i.e., boredom or disinterest) was induced. In contrast, the IGD group, which had a high interest in game stimuli, induced a sensitization process (i.e., craving). Previous studies related to brain activity have reported EEG responses in the pure resting state. (1) The beta power in the IGD group was higher than that in the HCs group [82]. (2) Patients with IGD showed increased resting-state EEG in slow-wave activity, such as delta and theta, compared with HCs [94]. The results of previous studies were inconsistent, but their findings on the delta, theta, and beta powers were consistent with the results of the present study. These results suggest that cortical activity is enhanced by the repeated or prolonged presentation of a stimulus. Generally, strict protocols are more distinctive between individuals and reproducible compared to those without tasks (i.e., resting-state) [95]. This suggests that the method proposed here can better induce electrophysiological responses in IGD groups than that proposed in previous studies and helps in distinguishing IGD from HCs. As mentioned above, because delta and beta powers are strongly related to behavioral inhibition and the reward circuit, our results can be interpreted as maintaining the dysfunction of behavioral inhibition in the prefrontal area and activation of the reward circuit, even after exposure to repeated stimuli.

In this study, we attempted to develop a quantitative method for the measurement of IGD and provide evidence to confirm its suitability for this purpose. If a quantitative diagnosis can be developed, it can have positive effects, such as (1) providing feedback to doctors or therapist to treat symptoms, (2) allowing therapeutic restrictions on playing games to be rationally based on IGD level (i.e., time spent playing the games), and (3) decreasing the development of IGD in high-risk groups by early measurement of IGD.

This study had several limitations. (1) Because additional groups were not considered in the experimental design of this study, the pattern of EEG features found in this study may be related to other behavioral addictions as well. IGD is also associated with depressed mood, anxiety, psychiatric disorders, attention deficit hyperactivity disorder, and obsessive-compulsive disorder. The possible comorbidity of IGD with other mental disorders should be considered when determining electrophysiological features of IGD. Thus, it cannot be conclusively stated that the findings of this study can distinguish IGD from other addictions or mental diseases; our findings require confirmation through further research. (2) Only male participants were included in this study because male adolescents tend to invest more time in playing games than female participants [53, 96]. The occurrence of IGD is higher in men than in women; however, IGD is also often reported in women. Thus, female participants should be considered in future studies on IGD. (3) The experiment was conducted based on a VR environment. None of the participants had any VR experience prior to the experiment. Therefore, this inter-participant difference from the VR-effect might be low. Although some participants could adapt to VR better than others, Pöhlmann et al. [97] reported that the discomfort from VR caused different effects such as illusion strength. This point is known to cause inter-participant differences according to degree of discomfort [97]. Therefore, the feelings of the participant after the VR experiment should be considered. (4) We believe that repeated presentation stimuli can give rise to different processes of habituation or sensitization depending on the person’s interest in stimuli of gaming and neutral video, further enhancing cortical activity. However, while watching both gaming and neutral video, it is possible that the person suffering from IGD is unknowingly also addicted to screen (TV, monitor, or mobile) regardless of the content type being watched. To clarify this issue, it is essential to separately compare pre- and post-resting state EEGs when neutral and gaming videos are being watched.

## Data Availability

The datasets generated and/or analysed during the current study are available in the devhaji / IGD_datasets repository (github), [https://github.com/devhaji/IGD_dataset/blob/main/README.md].
